# An evaluation of porcine epidemic diarrhea virus survival in individual feed ingredients in the presence or absence of a liquid antimicrobial

**DOI:** 10.1186/s40813-015-0003-0

**Published:** 2015-07-09

**Authors:** Scott Dee, Casey Neill, Travis Clement, Aaron Singrey, Jane Christopher-Hennings, Eric Nelson

**Affiliations:** 1 Pipestone Applied Research, Pipestone Veterinary Services, 1300 Box 188 Hwy 75 S, 56164 Pipestone, MN USA; 2 Animal Disease Research and Diagnostic Laboratory, South Dakota State University, Brookings, SD USA

**Keywords:** Porcine, Epidemic, Diarrhea, Virus, Antimicrobial, Feed, Ingredient, Bioassay, Formaldehyde

## Abstract

**Background:**

Contaminated complete feed and porcine plasma are risk factors for PEDV introduction to farms and a liquid antimicrobial has been proven useful for reducing risk. This study provides information on the survivability of PEDV across common swine feed ingredients in the presence or absence of the liquid antimicrobial.

**Results:**

Eighteen ingredients commonly included in commercial swine diets were selected, including 3 grain sources (corn, soybean meal (SBM), dried distillers grains with solubles (DDGS)), 5 porcine by-products (spray-dried plasma, purified plasma, intestinal mucosa, meat and bone meal and red blood cells (RBCs)), 3 vitamin/trace mineral (VTM) mixes (sow, nursery, finishing), 2 fat sources (choice white grease and soy oil), 3 synthetic amino acids (lysine HCL, D/L methionine, threonine), as well as limestone and dry choline chloride. Complete feed and stock PEDV served as controls. Thirty grams of each ingredient were inoculated with 2 mL PEDV. A matched set of samples were treated with the formaldehyde-based liquid antimicrobial SalCURB® (LA). All samples (*n* = 320) were stored outdoors under winter time ambient conditions for 30 days. Samples were submitted on 1, 7, 14 and 30 days post-inoculation (DPI) and tested by PCR and virus isolation (VI). All VI-negative samples were tested by swine bioassay. Viable PEDV was detected by VI or swine bioassay at 1, 7, 14 and 30 DPI from SBM, DDGS, meat & bone meal, RBCs, lysine HCL, D/L methionine, choice white grease, choline chloride, complete feed and stock virus control and at 7 DPI in limestone and at 14 DPI in threonine. Supplementary testing of complete feed and SBM indicated viable virus out to 45 and 180 DPI, respectively. All other samples were negative by VI and bioassay. In contrast, treatment with LA inactivated PEDV across all ingredients on 1 DPI and induced RNA reduction over time.

**Conclusions:**

Under the conditions of this study, PEDV viability in feed was influenced by ingredient with extended survival in SBM. Furthermore, LA treatment rendered virus inactive, independent of ingredient type.

## Background

Porcine epidemic diarrhea virus (PEDV) is an enveloped single-stranded positive sense RNA virus belonging to the Order *Nidovirales*, the family *Coronaviridae* and the genus *Alphacoronavirus* [1]. Following detection in the US swine population during May, 2013 the virus spread rapidly across the country [2]. Initial risk factors proposed for the spread of PEDV between herds included infected pigs, contaminated transport and aerosols [3–5]. PEDV infection of nursery swine results in reduced performance and fecal shedding for out to 24 day post-infection (DPI) [3]. In regards to the role of contaminated transport vehicles, data collected from the US suggests that collection points, such as harvest facilities and livestock auction markets are contaminated and can be a source of contamination of transport vehicles that return to pig farms [4]. Finally, PEDV RNA has been detected by PCR in aerosol samples out to 16 km from infected swine facilities and under experimental conditions may contain viable PEDV [5]. However, while pig-to-pig transmission of PEDV has been proven [3], controlled transmission studies providing proof of concept regarding the role of aerosols and transport have not been published at this time. In 2014, new risk factors for PEDV were identified: contaminated feed and feed ingredients. Initial reports indicated the ability of PEDV to survive in dry feed for 7 days and in wet feed for 28 days when stored at room temperature [6]. Proof of concept that contaminated complete feed could serve as a route of PEDV transmission to naïve pigs was published [7], with a follow-up report identifying spray-dried porcine plasma as a possible ingredient-specific risk [8]. Both of these studies collected contaminated feed material from farms or feed suppliers and performed controlled challenge studies involving naïve pigs. In both cases, use of swine bioassay demonstrated infection in naïve piglets following ingestion of contaminated feed bin material [7] or commercial plasma [8], therefore raising awareness of these risks. Recently, transmission of PEDV via ingestion of contaminated complete feed has been repeated and the minimum infectious dose calculated at 5.6 x 10^1^TCID_50_/mL (Ct = 37) [9].

In regards to management strategies to reduce the risk of PEDV spread between herds, protocols of transport sanitation and air filtration have been validated using standard approaches [Dee S, unpublished data, 2013–2015]; however, the means to biosecure feed is a new paradigm for the swine industry. Recently, a liquid antimicrobial (LA) product containing formaldehyde and propionic acid has been proposed as a means to mitigate risk (SalCURB®, Kemin Industries, Des Moines, IA, USA) [10]. This study demonstrated the ability to prevent PEDV infection in pigs consuming contaminated complete feed treated with LA [10]. However, information is not currently available regarding PEDV survival in other ingredients in swine diets or whether LA treatment can inactivate PEDV at the ingredient level. Therefore, a study was conducted to measure PEDV viability across a panel of ingredients commonly encountered in swine diets in the presence or absence of a LA presently used to maintain feed and feed ingredients as *Salmonella-*negative for up to 21 days. It was hypothesized that while PEDV survival is ingredient-specific, LA would be efficacious, independent of ingredient type.

## Results

### Sample size

A total of 320 feed ingredient samples were used for this study.

### PCR

**Fig. 1:**
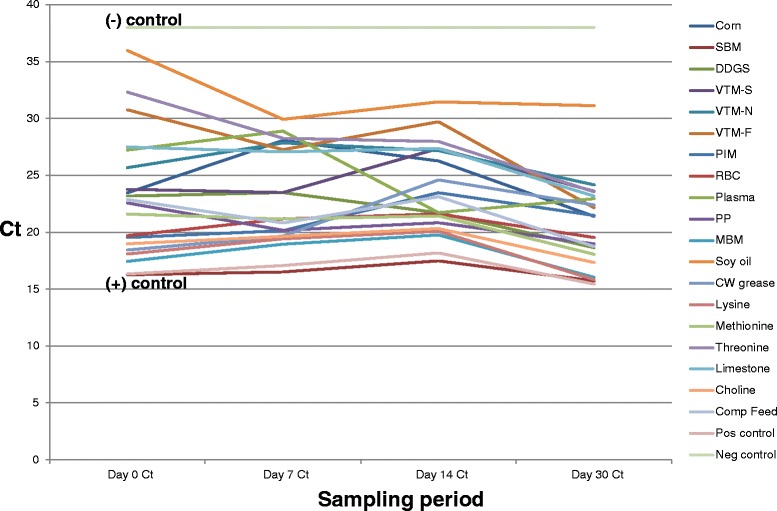
Change in mean PEDV Ct levels of non-treated ingredients throughout the sampling period. This figure depicts the change over time in Ct values in the non-treated (saline placebo) ingredient samples collected during the study period. All ingredients were determined to be PCR-negative prior to inoculation. After PEDV inoculation, Ct levels ranged across ingredients from a low of 16.26 (SBM) to a high of 35.98 (soy oil). Note the consistent trend of Ct across the ingredient panel, indicating that PEDV quantity in all ingredients is remaining relatively constant over time, as expected in the absence of intervention

**Fig. 2:**
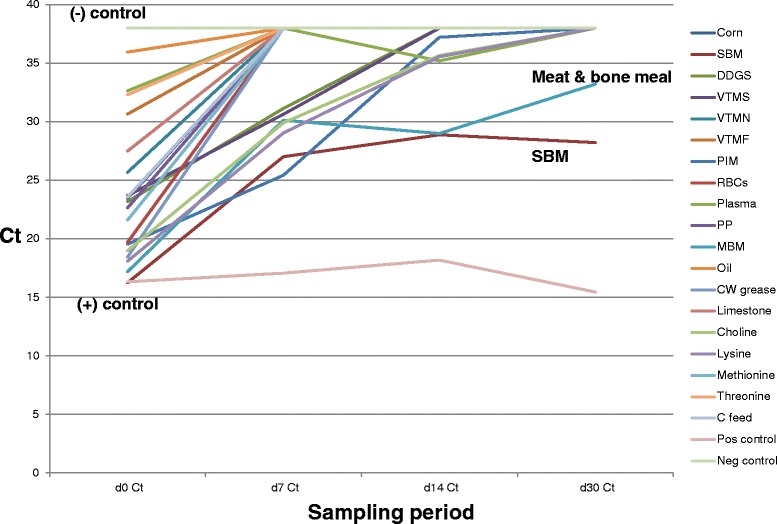
Change in mean PEDV Ct levels of liquid antimicrobial treated ingredients throughout the sampling period. In contrast to Fig. 1, this graph depicts the change over time in Ct values following treatment with LA. Note the consistent trend of Ct across the ingredient panel in the direction of the complete feed negative control (Ct = 38), indicating that PEDV quantity in all ingredients is decreasing over time. At day 30PI, 16 of the 18 selected ingredients have become PCR-negative, with the exception of SBM (Ct = 28.22) and meat and bone meal (Ct = 33.28). In addition, all ingredients were VI-negative on day 1 following LA application

All feed ingredient samples were PCR negative on day 0 of the study. Successful PEDV inoculation was confirmed, as all day 1 samples were PCR-positive. Results of PCR testing of LA treated and non-treated ingredients on 1 and 30 DPI are summarized in Table 1 and trends in Ct levels of treated and non-treated ingredients over the 30 day period are displayed in Figs. 1 and 2. The mean Ct of treated samples was 23.74 (SD = 5.9) on 1 DPI and 37.23 (SD = 2.5) on 30 DPI. Only SBM (Ct = 28.22) and meat and bone meal (Ct = 33.21) remained PCR positive at 30 days PI, all other treated samples had Ct values of ≥38 indicating PEDV RNA was not detected, including the treated positive control complete feed samples. When analyzed by *t*-test, the difference in mean Ct of treated ingredients was significant at *p* < 0.0001. In contrast, mean Ct values across non-treated ingredients on day 1 averaged 23.46 (SD = 5.5) and 20.78 (SD = 3.8) on day 30. When analyzed by *t*-test, this difference was not significant at *p* = 0.1143. Finally, when analyzed by *t*-test, the mean Ct of 30 DPI treated samples (Ct = 37.23) was significantly different (*p* < 0.0001) than the mean Ct of 30 DPI non-treated samples (20.78).

**Table 1:** Change in mean Ct of liquid antimicrobial treated ingredients, non-treated ingredients and controls over the course of the study period

Sample	Mean Ct day 1 PI	Mean Ct day 30 PI
LA treated ingredients	23.74	37.23
(+) Control treated complete feed	23.57	>38
(−) Control complete feed	>38	>38
(+) Control virus stock	16.45	15.45
Non-treated ingredients	23.46	20.78
(+) Control complete feed	22.87	18.75
(−) Control complete feed	>38	>38
(+) Control virus stock	16.33	16.45

### Virus isolation

Viable PEDV was detected on 1 DPI and all sampling days (7, 14 and 30 DPI) from non-treated SBM, DDGS, RBCs, meat and bone meal, lysine HCL and D/L methionine. Select samples of non-treated choice white grease (1 and 7 DPI), threonine (1, 7 and 14 DPI) and limestone (1 and 7 DPI) were also VI positive (Table 2). In contrast, all non-treated samples of corn, the 3 VTMs, spray-dried plasma, purified plasma, intestinal mucosa and dry choline chloride were VI negative on all 4 sampling days (Table 3). Both the complete feed positive control samples and the virus stock controls were VI positive on all sampling days. All complete feed negative control samples and all LA treated ingredients were VI negative across all sampling points.

**Table 2:** Summary of feed ingredients which contained viable PEDV as detected by virus isolation

Ingredient	Mean	Mean	Mean	Mean
	Ct/FFU titer	Ct/FFU titer	Ct/FFU titer	Ct/FFU titer
	Day1 PI	Day 7 PI	Day 14 PI	Day 30 PI
SBM	16.24/225,000	16.51/170,000	17.48/10,000	15.72/15,360
DDGS	23.17/17,500	23.29/9600	21.71/80	18.65/40
RBCs	19.70/160,000	21.18/25,000	21.61/3540	19.53/500
Meat & bone meal	17.44/25,325	18.95/25,600	19.76/1920	16.04/1320
Choice white grease	18.46/700	19.66/140	24.60/negative	22.60/negative
Lysine HCL	18.08/40	19.42/40	20.09/40	15.83/40
D/L Methionine	21.60/20,000	21.16/12,800	21.44/350	18.06/240
Threonine	29.31/40	28.27/40	27.98/40	23.55/negative
Limestone	27.49/40	27.06/40	27.37/negative	23.17/negative
(+) Control complete feed	22.87/750	20.81/500	18.40/640	18.75/150
(−) Control complete feed	>38/negative	>38/negative	>38/negative	>38/negative
Virus stock	16.34/200,000	17.35/200,000	18.40/12,800	18.95/5120

Ingredient: Two 30 g replicates per ingredient

Mean Ct/FFU titer: Mean Ct value and FFU/mL across the 2 samples per ingredient

**Table 3:** Summary of feed ingredients which did not contain viable PEDV as detected by virus isolation

Ingredient	Mean	Mean	Mean	Mean
	Ct/FFU titer	Ct/FFU titer	Ct/FFU titer	Ct/FFU titer
	Day1 PI	Day 7 PI	Day 14 PI	Day 30 PI
Corn	23.46/negative	28.08/negative	23.36/negative	21.40/negative
VTM-sow	23.77/negative	23.49/negative	27.37/negative	23.60/negative
VTM-nursery	25.67/negative	27.85/negative	27.18/negative	24.16/negative
VTM-finisher	30.77/negative	27.27/negative	29.71/negative	22.15/negative
Plasma	20.01/negative	21.18/negative	23.74/negative	23.48/negative
Purified plasma	22.08/negative	20.17/negative	20.84/negative	18.97/negative
Intestinal mucosa	19.55/negative	20.13/negative	20.84/negative	23.48/negative
Choline chloride	18.96/negative	19.63/negative	20.32/negative	15.74/negative
Soy oil	35.98/negative	29.93/negative	31.46/negative	31.13/negative

Ingredient: Two 30 g replicates per ingredient

Mean Ct/FFU titer: Mean Ct value and FFU/mL across the 2 samples per ingredient

### Swine bioassay

Samples selected for swine bioassay testing consisted of non-treated ingredients which were PCR positive/VI negative on 7, 14, and 30 DPI. This included corn, all 3 VTMs, intestinal mucosa, soy oil, choline chloride, spray-dried plasma, purified plasma and SalCURB® -treated ingredients. In addition, ingredients which VI-positive on 7 DPI but VI negative on 14 and 30 DPI (choice white grease and limestone) and threonine (VI negative 30 DPI) were tested as well. Following completion of the bioassay, viable PEDV was only detected in piglets in the choline unit and the choice white grease unit. Affected animals displayed evidence of mild diarrhea, shed PEDV in feces and samples of small intestine were PCR-positive at necropsy. All other piglets inoculated with the aforementioned feed ingredients, LA treated ingredients and the negative control piglets remained healthy and all rectal swabs and intestinal tract samples were negative by PCR. Results are summarized in Table 4.

**Table 4:** Summary of results of PCR (+)/VI (−) feed ingredients tested by swine bioassay

Ingredient	Samples pooled for inoculum	Ct of inoculum	Clinical signs/rectal swabs	PCR testing of small intestine
Corn	7, 14, 30 DPI	23.44	Negative	Negative
VTM-sow	7, 14, 30 DPI	24.82	Negative	Negative
VTM-nursery	7, 14, 30 DPI	26.39	Negative	Negative
VTM-finisher	7, 14, 30 DPI	26.38	Negative	Negative
Choice white grease	14, 30 DPI	23.21	Positive	Positive
Hydrolyzed intestinal mucosa	7, 14, 30 DPI	24.38	Negative	Negative
Soy oil	7, 14, 30 DPI	34.15	Negative	Negative
Threonine	30 DPI	28.27	Negative	Negative
Limestone	14, 30 DPI	28.07	Negative	Negative
Plasma	7, 14, 30 DPI	22.95	Negative	Negative
Purified plasma	7, 14, 30 DPI	23.46	Negative	Negative
Choline chloride	7, 14, 30 DPI	18.01	Positive	Positive
LA -treated	7, 14, 30 DPI	31.06	Negative	Negative
(−) Control	saline	>38	Negative	Negative

### Meteorological data

The mean temperature recorded during the 30 day sampling period encompassing day 1–7 was significantly lower (−18 °C) than the periods encompassing days 8–14 (−13 °C) and days 15–30 (−9 °C) as determined by ANOVA (*p* < 0.0001).

### Supplementary testing of SBM

**Fig. 3:**
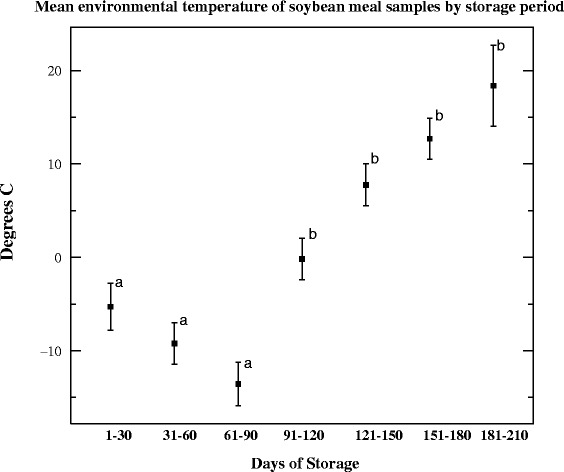
Mean environmental temperatures of soybean meal samples by storage period. This figure displays the mean and the 95 % confidence intervals associated with the project period (day 1–30 PI) and the 180 day supplementary testing period of soybean meal (SBM). Note the significant increase in mean temperature observed days 1–90 PI and days 91–210 PI. During the 210 day period, viral titers decreased (1–30 DPI), stabilized (31–150 DPI) then decreased in accordance with increasing temperature (151–180 DPI)

Based on the magnitude of the SBM titers at 30 DPI (mean = 15,360 FFU/mL), it was decided to continue to store these non-treated samples as described and test at 30 days intervals for an additional 180 days. At 180 DPI, mean Ct and mean FFU/mL across the 2 samples of SBM were 20.11 and 90 FFU/mL, respectively (Table 5). Throughout this period of time, the mean temperature was −0.13 °C (range = −25 °C to 22 °C, 95 % CI = −1.83 to 1.57 °C, median = 0.00 °C and SD = 11.7). Samples tested at 210 DPI were PCR-positive (21.84) but VI-negative. Differences in the mean temperature of sampling periods 1–90 DPI were significantly different (*p* < 0.0001) than those recorded during days 91–120 DPI versus 121–210 DPI (Fig. 3). As a control, the complete feed positive control was conducted and samples remained VI positive out to day 45 DPI (Ct 24.50, 160 FFU/mL).

**Table 5:** Change in Ct level and virus titer in soybean meal (SBM) samples during the 30-day study period and the 180-day supplementary testing period

	Mean Ct^a^	Mean titer
1 DPI	16.24	225,000
30 DPI	15.72	15,360
60 DPI	17.92	7500
90 DPI	18.83	6080
120 DPI	17.90	5000
150 DPI	18.52	6080
180 DPI	20.11	90
210 DPI	21.84	Negative

^a^Mean Ct/Mean titer: Mean Ct value and FFU/mL across the 2 samples per ingredient

## Discussion

The objective of this study was to measure PEDV viability across feed ingredients commonly encountered in swine diets in the presence or absence of a liquid antimicrobial treatment. The viability of food borne illnesses, such as *Salmonella sp*. have likewise been studied in feed and feed ingredients [11]. Based on the fact that ingredient processing procedures can successfully render PEDV inactive [12], this study operated under the premise of post-processing contamination of all ingredients. Under the conditions of this study, our results suggest that PEDV survival is ingredient-specific, as 50 % of the non-treated ingredients harbored viable PEDV over some period of time while the remaining non-treated ingredients were VI and bioassay negative at 7, 14 and 30 DPI. The extended survivability of PEDV in SBM was a novel observation and may have been influenced by environmental temperature, based on the observed decrease in titer as temperatures increased over time. In addition, other novel information from this study included the recovery of viable PEDV from DDGS, 3 synthetic amino acids and dry choline chloride (all non-treated). A by-product of the ethanol industry, wet DDGS are stored outside/uncovered while dry DDGS are stored in open warehouses, raising the risk of post-processing contamination. As they are widely used in swine diets and their turnover at mills is short (1–2 days), if contaminated, as under the conditions of our study, virus may remain viable and the ingredient could pose a risk to complete feed during mixing. Similarly, the observed variability in PEDV survival across the 3 amino acids raises the question of whether ingredient chemistry, for example, D/L methionine, a sulfur-containing amino acid, may have influenced titer magnitude and duration. In addition, the detection of viable virus in both synthetic amino acids and choline chloride raises the awareness of both domestic risk and transboundary risk, due to their widespread global trade.

Just as intriguing was the inability of viable PEDV to be recovered from certain ingredients, such as spray-dried porcine plasma, particularly since infection of naïve piglets through the ingestion of PCR-positive porcine plasma has been published [8]. Surprisingly, we were not able to reproduce these results, despite employing two viability assays and purposely inoculating plasma samples with PEDV at a Ct of 16.34 (400,000 FFU/total dose), a viral load far greater than previously published [8]. Even at day 1 PI, we were not able to recover viable PEDV by VI and samples collected at 7, 14 and 30 DPI were negative by bioassay. Follow-up questioning confirmed that the plasma had not been chemically altered at the mill. While negative results were obtained with intestinal mucosa samples and purified plasma, the risk of other by-products, such as meat and bone meal and RBCs continue to raise awareness that certain by-products, if contaminated, may harbor viable PEDV for extended periods.

Similar to plasma, no evidence of viable PEDV was recovered from the 3 different VTMs tested. This is interesting as international shipments of VTMs were originally considered a possible source of PEDV introduction to the US. Finally, in regards to the management of “high risk” ingredients, under the conditions of this study, the formaldehyde-based liquid antimicrobial successfully reduced viral load and inactivated PEDV, independent of ingredient. These data confirm previously published reports of the efficacy of liquid antimicrobial biosecurity of PEDV-contaminated complete feed [10].

As with all research studies, this project had its share of acknowledged strengths and limitations. Regarding the strengths of the study, we selected 18 ingredients commonly found in commercial swine diets and designed an experiment where the only variables in the study were the individual ingredients, and the presence or absence of the liquid antimicrobial. Specifically, we inoculated equal amounts of each ingredient with an equivalent quantity of virus in an attempt to mimic published viral loads associated with field cases of PEDV in feed. All ingredients were stored in identical containers, exposed to the same environment, sampled over periods representative of mill turnover times and tested in a single laboratory, involving consistent, trained personnel and validated assays for the detection of PEDV. We organized samples in a manner to prevent cross-contamination by insuring that the individual sample storage containers were never opened from the time of PEDV inoculation until testing occurred at the laboratory. While this may have resulted in some variability of the PCR assay since a new sample set was submitted each time, the fact that our negative control samples remained free of contamination throughout the entire project validated these protocols. Finally, we used multiple metrics (PCR, VI and bioassay) to document viral load across a total of 320 samples. At the time of this writing, this is the only such study we are aware of that has used this approach to assess PEDV viability in a large variety of ingredients.

In regards to limitations, while we did involve the use of a large number of samples, we were limited to 18 ingredients and we only conducted 2 replicates per ingredient. Furthermore, these results were derived from gram quantities of feed and may not directly equate to the vast quantity of tonnage used in actual swine production or feed manufacturing facilities. The bioassay protocol, while helpful at identifying ingredients containing low levels of virus presented limitations, was limited by cost thereby requiring pooling across sample days, i.e., 7, 14 and 30 DPI, instead of the testing of daily samples. Despite the need to pool samples, the Ct values across tested inoculums ranged from 22.95 to 31.06, clearly indicative of a high quantity of PEDV. Finally, the fact that the samples were stored under a specific set of environmental conditions for a defined period does not allow for extrapolation of results across other climates or over longer periods of time, with the exception of the supplementary testing of SBM and complete feed.

In closing, based on the information from this study, a theory to explain how PEDV infection of a swine farm via contaminated feed may actually occur in the field can now be proposed, based on the hypothesis that infection via contaminated feed is a multi-factorial event:

1. Ingredients capable of harboring viable PEDV for an extended period become contaminated.
2. Contaminated ingredients then contact a supportive matrix (SBM) during the preparation of complete feed.
3. Delivery of PEDV-positive feed introduces the virus to the farm.

While further work is needed, including replication under varying conditions of season, differing viral loads, a larger number of ingredient types, and alternative intervention methods, the risk of contaminated feed and feed ingredients as a vehicle for PEDV has now been well established. With this information, veterinarians and feed industry experts can now begin to target interventions to reduce risk. In addition, increased efforts at the mill level, focusing on improved biosecurity of facilities and feed transport vehicles may also reduce risk with the end result being a lowered incidence of feed-related PEDV cases across the national herd. Finally, this paper provides the first proof of concept data that PEDV can survive for extended periods in select feed ingredients. We hope the new information generated from this project improves the understanding of pathogen risk through feed at both the domestic and international levels, thereby stimulating further research efforts in this critical area.

## Conclusions

The results of this study indicate that PEDV viability in feed appears to be influenced by ingredient type, with extended survival reported in SBM. Furthermore, formaldehyde-based liquid antimicrobial treatment rendered virus inactive, independent of ingredient type.

## Methods

### Processing of feed ingredients

A panel of 18 ingredients frequently found in commercial swine diets were selected for this study, including 3 grain sources (corn, soybean meal (SBM), dried distillers grains with solubles (DDGS)), 5 porcine by-products commonly used as auxiliary sources of protein (spray-dried plasma, purified plasma, intestinal mucosa (PepNS, Midwest Ag Enterprises, Marshall, MN US and TechMix LLC, Stewart, MN, US), meat and bone meal and red blood cells (RBCs)), 3 vitamin/trace mineral (VTM) mixes (sow, nursery, finishing), 2 fat sources (choice white grease and soy oil), 3 synthetic amino acids (lysine HCL, D/L methionine, threonine), as well as limestone and dry choline chloride. Ingredients were screened by PCR to insure a PEDV-negative status prior to use. The experiment was designed to evaluate viability of PEDV in ingredients over time; therefore, it was planned to sample each ingredient at 4 independent time periods: 1, 7, 14 and 30 days post-inoculation (DPI). As we were starting with PEDV-free ingredients, the day 1 sample would be used to confirm that contamination occurred and the extended sampling would be used to estimate the duration of survival over a 30-day period. The specific sampling times were selected based on estimated ingredient turnover time at mills and processing plants, with certain bulk macro-ingredients, such as SBM, DDGS having short turnover times, i.e., 1–2 days of storage, while bagged micro-ingredients, such as synthetic amino acids, dry choline chloride and VTMs, have longer periods (21–28 days) of storage (C. Neill, personal communication, 2014). To increase statistical power, it was planned to conduct 2 replicates of each ingredient with LA treatment and 2 non-treated (placebo) replicates of each ingredient. Therefore, sixteen 30 g samples of each of the 18 ingredients (*n* = 288) were added to individual freezer storage containers (Oxo Tot Baby Blocks, Oxo International, El Paso, TX, USA). Half of the samples of each ingredient (*n* = 144) were treated with 0.1 mL of LA (Kemin Industries, Des Moines, IA USA), based on an inclusion rate of 3 kg/t of complete feed. SalCURB® is a premix of aqueous formaldehyde solution 37 % (for maintenance of complete animal feeds or feed ingredients *Salmonella*-negative for up to 21 days) and propionic acid (as a chemical preservative for control of mold in feed or feed ingredients). While SalCURB® provides effective *Salmonella* control for up to 21 days, it is not approved for use by the U.S. Food & Drug Administration or the U.S. Department of Agriculture as a treatment for PEDV. The liquid antimicrobial was added to the designated 30 g samples using a tuberculin syringe. To promote proper mixing, the feed was stirred manually for 10 clockwise rotations and 10 counter-clockwise rotations using individual wooden applicator sticks per ingredient. The remaining 144 samples were treated using 0.1 mL of saline placebo and mixed in a similar manner.

Following treatment, all samples (30 g each) were inoculated with 2 mL PEDV (passage 18, Ct = 16.34, (400,000 FFU/total dose), approximately 4–5 logs/mL TCID_50_) and mixed as described. This quantity of PEDV was selected in an effort to provide a final mean Ct value in feed ingredient of approximately 25 (range = 19–30) following mixing, based on data from actual field cases of PEDV-contaminated feed (S. Dee, unpublished observations, 2014–2015), a challenge level used in published studies [7, 10]. Once prepared, samples were stored outdoors in large plastic covered totes (Rubbermaid Cleverstore, 92.5 l capacity, Newell Rubbermaid, Atlanta, GA, USA) with one tote containing LA treated samples and the other the non-treated samples. Samples were stored outdoors (state of Minnesota, site coordinates latitude 45^0^ 49’ N, longitude 95^0^ 33’ W) during winter time ambient conditions for a 30 day period (January 3–February 2, 2015). These conditions were selected based on previously published data indicating the ability of the virus to survive under a wide range of pH when stored at cold temperatures [13]. In addition, many of these ingredients are routinely stored outdoors, uncovered at processing plants and grain elevators, or in unheated milling facilities (S. Dee, unpublished observations, 2013–2015). Therefore, to maximize virus survival and to accurately represent “real-world” conditions found throughout the feed industry in the upper Midwest USA, storage under winter time ambient conditions was selected. During the study period, it was planned to remove 4 samples of each ingredient (2 LA treated, 2 non-treated) from their respective totes and submit for diagnostic testing on the designated (1, 7, 14 or 30) DPI. In other words, the same sample was not repeatedly opened and tested, but rather a new set of samples were submitted on each sampling day. Besides storage of ingredients in sealed individual containers, containers were stored in plastic bags by sample date, for example, the day 7 samples were bagged separately from day 14 samples which were bagged separately from the day 30 samples, etc. The comprehensive means of sample management would insure that all sample containers remained sealed from the time they were inoculated until the time they were tested at the lab, eliminating the risk of cross-contamination during storage and delivery to the laboratory.

### Controls

For the purpose of controls, 32 samples of complete feed were inoculated with PEDV (16 positive control samples) or saline (16 negative control samples) as per the ingredients. In addition, 8–10 mL samples of stock PEDV in minimum essential media (Difco, Detroit, MI, USA) served as stock virus controls. All controls were managed in an identical manner as the ingredients. Finally, as with the ingredients, controls were treated with LA or saline and submitted for diagnostic testing at 1, 7, 14 and 30 DPI.

### Diagnostic procedures

All diagnostic testing was conducted using protocols developed and validated by the South Dakota State University (SDSU) Animal Disease Research and Diagnostic Laboratory (ADRDL). Samples were submitted by code to the laboratory, so personnel were blinded as to day, treatment and ingredient type.

### Extraction of RNA

The MagMAX™ 96 Viral Isolation Kit (Life Technologies, Waltham MA, USA) was used to obtain viral RNA from the samples, as described in the instructions provided (1836 M Revision F). A 175-μl volume of sample was used for the extraction. The magnetic bead extractions were completed on a Kingfisher96 instrument (Thermo Scientific, Waltham MA, USA).

### Real-time PCR

A commercially available real-time, single tube RT-PCR multiplex assay for the detection of PEDV, porcine deltacoronavirus (PDCoV) and transmissible gastroenteritis virus (TGEV) was used in this study per kit instruction (Tetracore, Rockville, MD, USA). Briefly, 7 μl of the extracted RNA was added to 18 μl of the master mix. The one-step real-time RT-PCR amplification conditions started with 15 min at 48 °C, followed by 2 min at 95 °C. The final cycles consisted of 5 s at 95 °C and then 40 s at 60 °C (data collection step). The program was run for 38 cycles (Cycle time) with PEDV positive results indicated at ≤ 38 cycles. Positive and negative controls were included on each run. All amplification was completed on the ABI7500 instrumentation (Austin, TX, USA).

### PEDV stock virus propagation

For PEDV propagation, Vero 76 cells (ATCC CRL-1587) were maintained in MEM plus 10 % fetal bovine serum and antibiotics. Three-day old confluent monolayers of Vero 76 cells in 150 cm^2^ flasks were washed 3 times with serum free minimum essential media (MEM) prior to inoculation. Monolayers were infected at ~0.1 moi of PEDV in MEM containing 2 *u*g/ml TPCK treated trypsin, incubated at 37 °C for approximately 48 h until obvious CPE was apparent. Flasks were frozen at −80 °C until needed.

### Virus isolation

Once feed ingredient samples were tested for PEDV via PCR, the residual samples were tested for presence of viable virus. Samples were diluted in MEM containing 2 μg/ml TPCK-treated trypsin with a starting dilution of 1:2 and were two-fold serially diluted. Diluted samples were then added to washed confluent monolayers of Vero-76 cells in 96-well plates and incubated for 1 h at 37 °C. Plates were again washed and trypsin media replaced. After 24 h at 37 °C, plates were fixed with 80 % acetone and stained with FITC conjugated mAb SD6-29 to allow visualization of infected cells. Virus concentration was determined by calculating FFU/ml based on the number of fluorescent foci present in wells at selected dilutions using a previously published method adapted to PEDV [14]. Personnel reading the plates were blinded to the type of sample and the time of sampling.

### Swine bioassay

#### Facilities and source of animals

The purpose of the swine bioassay was to determine whether viable PEDV was present in any feed ingredient sample that had tested positive on PCR but negative on VI. This study was conducted in a Biosafety Level 2+ room at the Animal Resource Wing (ARW) at South Dakota State University. All procedures involving animals throughout the study were performed under the guidance and approval of the SDSU Institutional Animal Care and Use Committee. Animals (*n* = 24, 5–7 day old piglets) were sourced from a PEDV-naïve herd and were tested on arrival to the ARW via blood sampling and collection of rectal swabs from each pig. Prior to animal arrival, all rooms (walls, ceilings, floors and drains) were monitored for the presence of PEDV by PCR using sampling procedures previously described [8, 10]. Piglets were housed in one of 6 stainless steel gnotobiotic units measuring 0.6 m W x 1.2 m L x 0.6 m H. Units were divided into 4 semi-isolated housing units, allowing for 4 piglets per unit with individual feeding arrangements. Flooring consisted of an open weave rubberized mat on a perforated stainless steel grate raised 10 cm for waste collection. Each unit was covered with an inflatable 20 mil plastic canopy and fitted with 2 pair of dry-box gloves for feeding and procedures inside the canopy. Each canopy was secured and sealed with duct tape and ratchet straps to the unit. Ventilation was supplied by an electric fan maintaining sufficient positive pressure inside the canopy to keep the canopy inflated above the unit. Incoming and outgoing air to each unit was HEPA-filtered. Each unit was initially sterilized using 47 % aerosolized formalin, and allowed to dissipate for 2 weeks prior to introduction of the animals. All incoming and outgoing materials needed during the study (eg. swabs, injectable medication, bleeding supplies) were passed through an air-tight stainless steel port and sterilized using 5 % peracetic acid before entering or exiting the port.

#### Preparation of bioassay inoculum

The stainless steel unit served as the experimental unit; therefore, all 4 piglets in each unit received the same ingredient. To assess PEDV survivability under representative storage times, it was planned to test samples previously determined to be PCR positive and VI negative on days 7–30 DPI. For preparation of the inoculums, 60 g of each specific ingredient from 7, 14 and 30 DPI were pooled and mixed with 50 mL of sterile PBS in a 250 mL centrifuge tube, inverted 10 times to mix and vortexed for 2 min. The suspension was then centrifuged at 5,200 g for 15 min, supernatant decanted and tested by PCR prior to piglet inoculation. Each pig in the unit received 1 mL of the designated inoculum orally via syringe and observed for a 7 day period. To minimize the number of animals needed for the study, pigs that were confirmed negative after 7 DPI would be inoculated with a different ingredient. A negative control unit was included in the design, with these pigs receiving sterile saline PO.

### Piglet testing

Following inoculation, the PEDV status of each group of piglets was monitored [8, 10]. On a daily basis, ARW personnel inspected animals for clinical signs of PED and collected rectal swabs (Dacron swabs, Fisher Scientific, Franklin Lakes, NJ, USA) from each pig, starting with the negative control unit. Showers were taken upon entry to the rooms and room-specific coveralls, footwear, hairnets, gloves and P95 masks (3 M, St. Paul, MN USA) were worn. In addition, each room was ventilated individually and HEPA filtration for both incoming and outgoing air was employed per room. If clinically affected animals were observed, swabs of diarrhea and/or vomiting, in conjunction with the daily rectal swab were collected. Swabs were submitted to the SDSU ADRDL and tested by PCR. If PEDV was diagnosed in a specific unit, all animals were swabbed, humanely euthanized with intravenous sodium pentobarbital, the small intestinal tracts submitted for PCR testing, units were cleaned and sanitized as described and re-stocked with new piglets as needed.

### Meteorological trends during study period

Ambient temperature over the period of January 3 through February 2 was collected using Weather Underground (wunderground.com). Data encompassing time periods relative to each sampling point (day 1–7, day 8–14 and day 15–30) were described statistically. Additional temperature data were collected during the supplementary testing period of SBM (Fig. 3).

### Data analysis

Descriptive statistics, *T*-test and ANOVA were used to analyze data.

### Availability of supporting data

The data set (s) supporting the results of this article is included within the article.

## Abbreviations

PEDV: Porcine epidemic diarrhea virus
PED: Porcine epidemic diarrhea
Ct: Cycle threshold
PCR: Polymerase chain reaction
ARW: Animal resource wing
MEM: Minimal essential media
SBM: Soybean meal
DDGS: Distillers dried grains with solubles
RBCs: Red blood cells
LA: Liquid antimicrobial
